# Single-cell adhesivity distribution of glycocalyx digested cancer cells from high spatial resolution label-free biosensor measurements

**DOI:** 10.1016/j.mbplus.2022.100103

**Published:** 2022-02-05

**Authors:** N. Kanyo, K.D. Kovács, S.V. Kovács, B. Béres, B. Peter, I. Székács, R. Horvath

**Affiliations:** a Nanobiosensorics Laboratory, ELKH EK MFA, Budapest, Hungary; b ELTE Eötvös Loránd University, Department of Biological Physics, Budapest, Hungary

**Keywords:** Glycocalyx digestion, Cell adhesion, Single-cell, Biosensor, Population distribution, Subpopulations

## Abstract

The glycocalyx is a cell surface sugar layer of most cell types that greatly influences the interaction of cells with their environment. Its components are glycolipids, glycoproteins, and oligosaccharides. Interestingly, cancer cells have a thicker glycocalyx layer compared to healthy cells, but to date, there has been no consensus in the literature on the exact role of cell surface polysaccharides and their derivatives in cellular adhesion and signaling. In our previous work we discovered that specific glycocalyx components of cancer cells regulate the kinetics and strength of adhesion on RGD (arginine-glycine-aspartic acid) peptide-coated surfaces [1]. Depending on the employed enzyme concentration digesting specific components both adhesion strengthening and weakening could be observed by monitoring the averaged behavior of thousands of cells. The enzyme chondroitinase ABC (ChrABC) was used to digest the chondroitin-4-sulfate, chondroitin-6-sulfate, and dermatan sulfate components in the glycocalyx of cancer cells. In the present work, a high spatial resolution label-free optical biosensor was employed to monitor the adhesivity of cancer cells both at the single-cell and population level. Population-level distributions of single-cell adhesivity were first recorded and analyzed when ChrABC was added to the adhering cells. At relatively low and high ChrABC concentrations subpopulations with remarkably large and weak adhesivity were identified. The changes in the adhesivity distribution due to the enzyme treatment were analyzed and the subpopulations most affected by the enzyme treatment were highlighted. The presented results open up new directions in glycocalyx related cell adhesion research and in the development of more meaningful targeted cancer treatments affecting adhesion.

## Highlights

• A high spatial resolution label-free biosensor monitors the adhesivity of cancer cells.
• Chondroitinase ABC was added to the adhering cells to digest their glycocalyx.
• Population level distributions of single-cell adhesivity were first recorded and analyzed.
At relatively low and high concentration subpopulations were identified.
The found subpopulations have remarkably large and weak adhesivities.

## Introduction

Cell adhesion is fundamental to life. Except for some cell types, for example, immune cells [2], [3] and circulating tumor cells [4], [5], cells need to adhere to the extracellular matrix or to the surface of another cell to survive. The adhesion is influenced by both intracellular and extracellular factors such as the cytoskeleton, membrane-bound adhesion proteins, and elements of the glycocalyx [1], [5], [6], [7], [8]. The glycocalyx is a multifunctional, carbohydrate-rich layer covering the cell surface. Its main components are proteoglycans, glycolipids, and glycoproteins [9]. Enzymatic removal of either of its components significantly affects the function and properties of the glycocalyx [10]. Importantly, depending on the cellular environment (e.g. cationic content and concentration, pH) and various external factors (e.g. shear stress), its composition dynamically changes [10], [11]. Proteoglycans are the major backbone molecules of the glycocalyx, consisting of a core protein (syndecans and glypicans) and its associated glycosaminoglycan (GAG) (heparan sulfate (HS) and hyaluronic acid (HA), chondroitin sulfate (CS), keratan sulfate, dermatan sulfate) chain. Glycocalyx components can be categorized according to the type of central protein and its GAG chains [10], [12]. The glycocalyx acts as a buffer layer between the cell membrane and the ECM, performing an important mechanical function [13]. The biological consequences of specific glycocalyx characteristics have been mainly studied for endothelial cells [10], [12], [14], [15], [16], [17].

The modified glycocalyx is actively involved in diseases that cause vascular symptoms, such as low instantaneous red blood cell volume in the capillaries [10]. Enzymatic modification of some proteoglycans of endothelial glycocalyx significantly increases capillary hematocrit and decreases blood flow resistance in the microvascular network [11]. Another study looked at the coronary glycocalyx of pigeon cork, associated with a thinner coronary glycocalyx with a higher susceptibility to disease and the glycolytic effect of diets high in fat and cholesterol, leading to arterial disease [12,61]. In another endothelial cell study, rat fat pads were examined. Using confocal microscopy, it was observed that the glycocalyx structure collapses with the digestion of certain GAG components (HS, CS, HA). Removal of HS or HA did not result in cleavage or collapse of the remaining components. However, simultaneous removal of CS and HA with chondroitinase reduced the adsorbed albumin amount, although the effect was not large [18]. Based on immunofluorescence analysis by Cancel et al., glycocalyx detachment has been associated with endothelial dysfunction and inflammation in athero-prone regions of the vasculature [19]. Mensah et al. have reported that the interaction of circulating tumor cells and vascular endothelial cells plays a significant role in the development of tumor metastatic diseases, and endothelial glycocalyx is also involved in these processes. It was found during wheat germ agglutinin (WGA) -labeled staining of glycocalyx that 50% of the glycocalyx is reduced by disturbed flow, resulting in the entry of circulating tumor cells into the endothelium, which is the first step in secondary tumor formation [20]. In another notable study, Harding et al. examined the state of extracellular glycocalyx at healthy and disruptive flow conditions in cell and mouse models. It was observed that in the healthy case, a thick glycocalyx covered the surface of the endothelium, whereas the expression of glycocalyx was significantly reduced under disturbed flow conditions. Regeneration of degraded glycocalyx may be a potential therapeutic approach to alleviate vascular disease [21].

The structure and composition of the glycocalyx layer of cancer cells differ greatly from that of healthy cells. For example, 95% of breast cancer cells have a modified glycocalyx composition or structure that also reshapes their function compared to the glycocalyx of a healthy cell [22]. Tumor cells are characterized by a thicker, high-density glycocalyx, which generates increased tension in the cell membrane, promotes the formation of integrins into clusters, alters the mechanical properties of the tissue, and increases the likelihood of developing a more cancerous phenotype [12], [13]. The increased flow shear stress enhances the secretion of metalloproteinase into the extracellular matrix, which facilitates tumor migration. The decomposed extracellular matrix is more permeable, and eventually, the tumor moves out of the tissue and enters the vascular system [23]. Glycocalyx has also a crucial role in receptor-ligand interactions between cancer cells and their environment, allowing intra- and extravasation. In addition, the composition and structure of glycocalyx greatly influence the transport and survival of circulating tumor cells in the bloodstream [4]. Clinical studies have shown that high glycoprotein levels are abundantly expressed in circulating tumor cells and have an altered glycosyltransferase expression profile in patients with advanced breast cancer. The thick glycocalyx is a characteristic of tumor cells that promotes metastasis even on soft substrate surfaces by mechanically enhancing cell surface receptor function, even in microenvironments with minimally adhesive conditions [5].

Under in vitro conditions, cell adhesion proceeds by passive adsorption to the surface, where initial contact may be established by cellular glycocalyx, followed by binding, the formation of focal adhesions, and cell migration or proliferation [5], [7], [8]. However, the exact definition of these processes, especially at the single-cell and population distribution level [24], is missing. There is a heavy need for label-free in situ technologies measuring these processes in intact living cells, preferably with single-cell resolution in a high-throughput manner.

There are several methods for monitoring the adhesion of living cells [25]. One of the most promising is the high-resolution resonant waveguide grating (RWG)-based optical biosensor. With this novel high-throughput technology, the adhesion of living cells can be monitored in real-time with extremely high resolution through a refractive index change induced by the accumulation of adhesion proteins inside the cell-substratum contact zone [26], [27], [28].

A common feature of optical techniques is that during their operation an optical electromagnetic wave interacts with the sample. A widely used example is the surface plasmon resonance (SPR) technology [29]. The sensitivity of interferometry-based methods is even higher and were recently demonstrated to monitor the real-time binding kinetics of ions to proteins [30], [31], [32]. With the help of optical waveguide biosensors, it is also possible to examine intact cells in situ [33]. Optical waveguide sensors have been successfully used in primary cell measurements to investigate changes in human monocyte adhesion as a function of serum concentration [34].

The physiological processes of the cells can be measured with an RWG using the convenient standard microplate-based format. The great advantage of the RWG sensor is that it measures the mass rearrangement inside the cells through the local refractive index change inside the cell-substratum contact zone. From this signal, various molecular-level processes can be inferred in real time, such as cellular signaling, division, or adhesion [1], [26], [27], [28]. Cell adhesion at the single-cell level can be well characterized by atomic force microscopy (AFM) [16], [35], [36]. However, the throughput of AFM is extremely low (5–10 cells/day). Therefore, it is not suitable to investigate population-level adhesion phenomena [25]. Much higher throughput can be reached with fluidic force microscopy or the single-cell level RWG biosensor employed in the present work [37].

In HeLa cervical cancer cells, the main GAG is chondroitin sulfate followed by heparan sulfate [38], and do not contain a significant amount of hyaluronic acid [18], [39], [40]. In our previous work, we discovered that different degrees of digestion of the HeLa glycocalyx by chondroitinase ABC (ChrABC) regulates the kinetics and strength of adhesion on RGD (arginine-glycine-aspartic acid) peptide-coated surfaces [1]. Depending on the employed concentration of ChrABC both adhesion strengthening and weakening could be observed. The effect was also examined in another cancer cell line (MCF-7) and in a healthy (preosteoblast MC3T3-E1) cell line, however, HeLa cells showed the greatest decrease in adhesion upon ChrABC treatment [1]. The phenomenon was investigated at the population average level, by employing 8000 cells inside the sensor wells. Moreover, by analyzing the real-time kinetic signals of adhesion, we could first determine the integrin-RGD binding constants in the presence of the glycocalyx components, in intact cells, without using any labeling [1].

In the present work, a high spatial resolution RWG biosensor was employed to monitor the adhesivity of cancer cells to reveal the population distributions of single-cell adhesivity. During the experiments, ChrABC was added to the adhering cells with various concentrations and the adhesivity was recorded. We found that the population distributions of cell adhesivity were well fitted by lognormal distributions. Enzymatic digestion of glycocalyx resulted in a broader population distribution with lower mean and median values. Furthermore, at relatively low and high ChrABC concentration subpopulations with remarkably large and weak adhesivity were also identified.

## Materials and methods

### Cell culture

HeLa cells were maintained in tissue culture polystyrene Petri dishes (Sarstedt, Germany) in a humidified incubator (37 °C, 5% CO_2_) in Dulbecco’s modified Eagle’s medium supplemented with 10% fetal bovine serum (S13665S181H, Biowest SAS, France), 4 mM L-glutamine (G7513, Merck, Germany), 100 U/ml penicillin and 100 μg/ml streptomycin mixture solution (Merck, Germany).

### Preparation of polymer solutions for coating the biosensor surfaces

The synthetic copolymers, poly(L-lysine)-*graft*-poly(ethylene glycol) (PLL-*g*-PEG, [PLL(20)-g(3.5)-PEG(2)]) (SZ42-28,SuSoS AG, Dübendorf, Switzerland) and its RGD-functionalized counterpart (PLL-*g*-PEG-DBCO-Mal)-CKK-(Acp)-(Acp)-(Acp)- GRGDS (hereafter PP-DBCO-R) were obtained as powders from SuSoS AG, Dübendorf, Switzerland (SZ43-74) [1], [41], [42], [43].

The materials were stored at −20 °C until use. Each powder was then dissolved in 10 mM 4-(2-hydroxyethyl)-1-piperazineethanesulfonic acid (HEPES, H3375 from Sigma-Aldrich Chemie GmbH, Munich, Germany) at pH 7.4 to make stock solutions with a concentration of 1.0 mg/ml and sterile filtered. Coating solution with different concentration of RGD motifs and PLL-*g*-PEG were prepared by mixing the two 1 mg/ml stock solutions in 1:1 rate, so 50% PLL-*g*-PEG-RGD (PPR) was obtained, then 30 μl of this mixture was added to the wells of Epic biosensor plate and incubated for 30 min at room temperature on a shaking machine. Reagent excess was removed by rinsing the surface three times with 20 mM HEPES to Hank’s balanced salt solution (HEPES HBSS, H8264 from Sigma-Aldrich Chemie GmbH, Munich, Germany), pH 7.4.

### Resonant waveguide grating optical biosensor with single-cell resolution

**Fig. 1:**
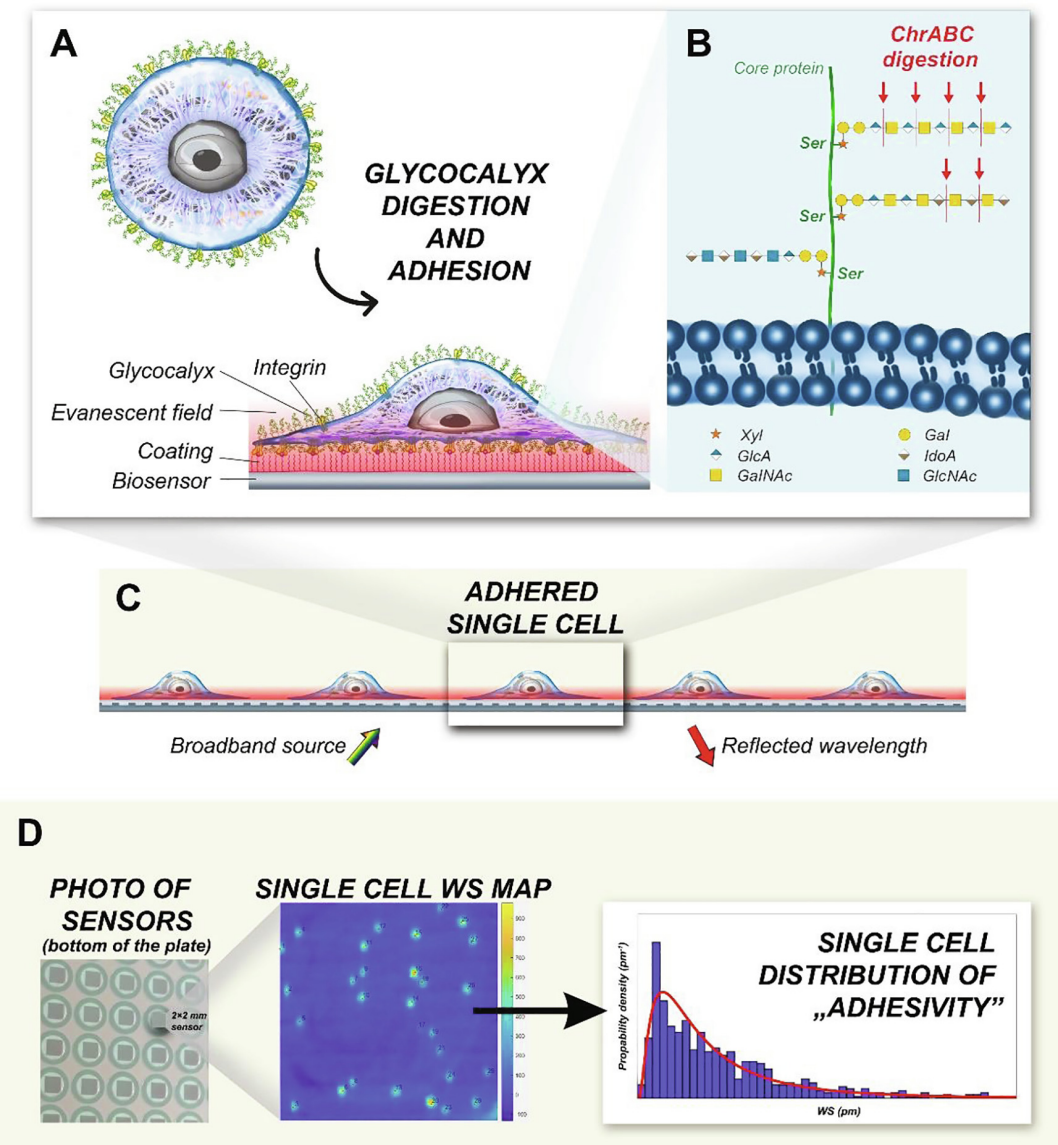
Schematic representation of the applied label-free method. A ChrABC enzyme digests the glycocalyx components of the HeLa cells. Using the evanescent field (red shadow area), the adhesion process of the digested cells on the PPR coated biosensor surface can be monitored in a label-free, real-time way. B Schematic illustration of the glycocalyx components. ChrABC cleaves dermatan sulfate, chondroitin 4-sulfate, and chondroitin 6-sulfate. It degrades polysaccharides containing (1–4)-β-D-hexosaminyl and (1–3)-β-D-glucuronosyl (or (1–3)-α-L-iduronosyl) linkages to disaccharides containing 4-deoxy-β-D-gluc-4-enuronosyl groups [1]. Cells adhere to the sensor surface that is illuminated from below (rainbow colored arrow) and only a certain resonant wavelength (red arrow) is reflected. The evanescent field (red area) penetrates the surface structures of the cell inside the cell-substratum contact zone. C With the RWG biosensor, even single-cells can be studied with high resolution. D The device works with 384-well microplates with 2×2 mm RWG biosensors in each well (left). The primary output of the device is the wavelength shift (WS) map in each well (middle). Individual cells can be easily identified, and the population-level distribution of cell adhesivity can be analyzed (right).(Note: parts of the figure (A,B and C) are adapted from ref. [1], [28]; use permitted under the Creative Commons Attribution 4.0 International License (http://creativecommons.org/licenses/by/4.0/)). (For interpretation of the references to colour in this figure legend, the reader is referred to the web version of this article.)

Epic Cardio (Corning Inc., USA), a label-free RWG-based optical biosensor, that records the kinetics of cell adhesion with single-cell resolution, was employed in the present work. The primary signal output of the instrument is the wavelength shift (WS) of the resonant wavelength which can excite the waveguide mode inside the optical waveguide sensor structure. The instrument records the WS image in every 3 s, where individual cells can be easily identified (see Fig. 1.). The maximum value of the WS signal corresponding to a given cell is defined as cell adhesivity in the present work. The WS signal is proportional to the cell mass per unit area inside the cell adhesion contact zone. Therefore, the WS signal is also a measure of the cell-substratum contact zone density of a given cell [25], [34]. Corning Epic cell adhesion 384 well microplates were employed during the measurements with a 2x2 mm^2^ RWG sensor area in each of the wells.

### Cell adhesion assay

Cell adhesion assay buffer was prepared by adding 20 mM HEPES to HBSS, the pH was adjusted to pH 7.4, and solution was filtered through a filter (0.22 μm). Upon surface modification, stable baselines were established in the wells with 20 µl of HEPES HBSS in the Epic Cardio instrument and after the baseline stagnant 20 µl of ChrABC, (C2905 Merck Germany) enzyme solution was added to the wells (control wells received the same amount of assay buffer), then 20 µl of cell suspension were added to the wells and biosensor responses were recorded for 2 h. Cells were prepared in the following way: following the cell detachment standard protocol using 0.05% (w/v) trypsin, 0.02% (w/v) EDTA solution, harvested cells were washed two times by centrifugation at 200×*g* for 5 min to remove the complete culture medium and cell pellet was re-suspended in 20 mM HEPES HBSS buffer. Cells were then counted in a hemocytometer and diluted to a final cell density of 45 cells/well.

## Results and discussions

Fig. 1 briefly summarizes the measurement setup and the evaluation protocol to obtain the population distributions of cell adhesivity. Individual cells can be easily recognized on the recorded WS image and the maximum WS signals corresponding to the individual cells were saved and analyzed. The upper left part shows the adhesion of a cell on the surface of the biosensor, which was previously coated with a synthetic polymer displaying the RGD peptide motifs. The evanescent wave-based optical biosensor measures the refractive index change close to the sensor surface, basically in a 150 nm thick surface layer corresponding to the cell-substratum contact zone. In this 150 nm thick layer, the binding of cell surface integrins to the RGD ligands can be detected.

The effect of glycocalyx elements on these interactions can be then monitored in a straightforward manner. To target specific components, ChrABC was used to digest the glycocalyx. This enzyme removes O-linked chondroitin sulfate-like glycosaminoglycans [44], [45], [46]. During the single-cell biosensor measurement care must be taken that the cells are not too close to each other for easy recognition of individual cells. In the present work, a maximum of about 45 cells were placed on a 2x2 mm^2^ biosensor surface.

**Fig. 2:**
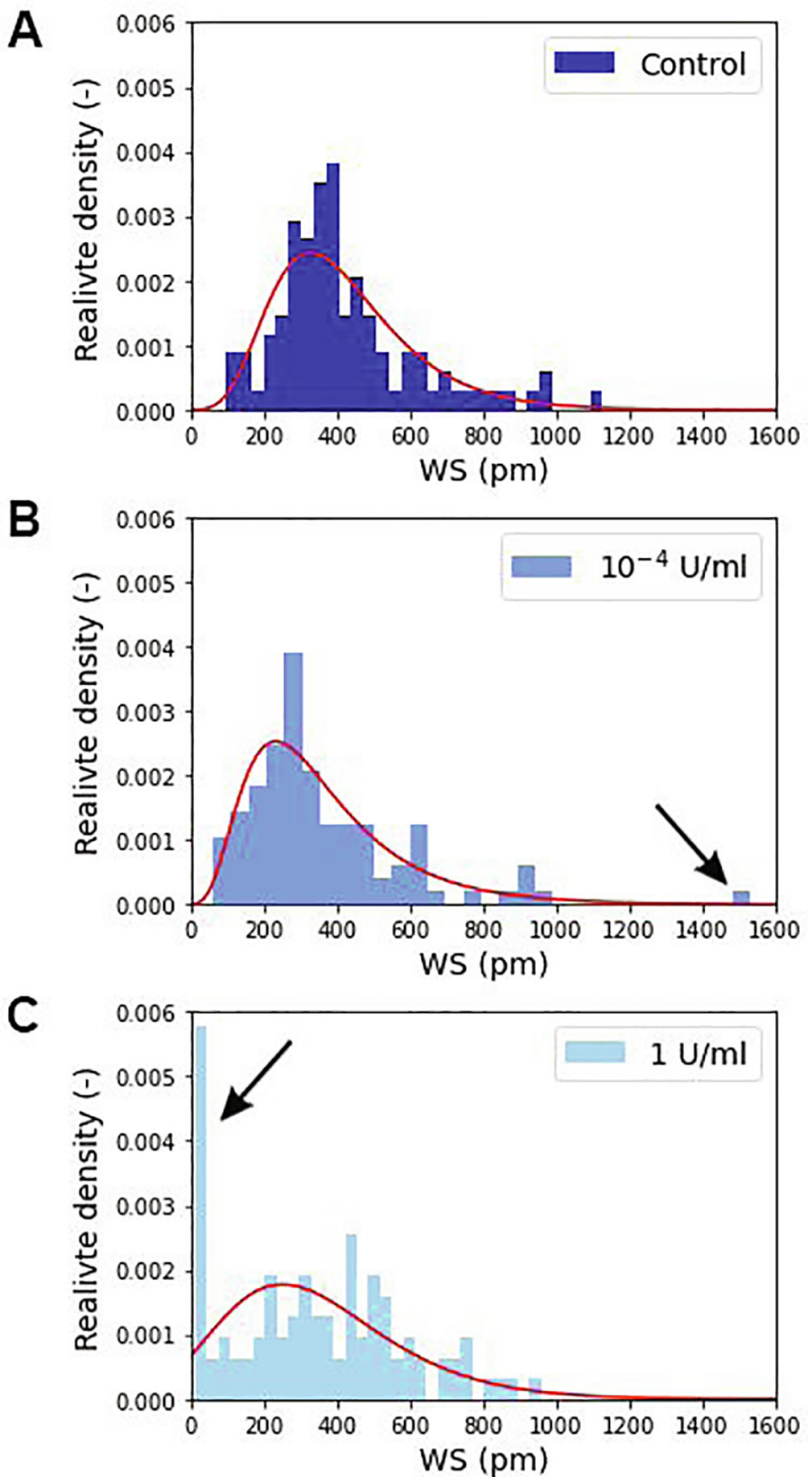
The measured population distributions of single-cell adhesivity and the corresponding lognormal fits (red lines) for the control population (A) and for the employed ChrABC concentrations (B,C). The larger the bin value the more cells adhere with the given WS signal. The black arrows indicate the new subpopulations appearing due to the enzyme treatments. (For interpretation of the references to colour in this figure legend, the reader is referred to the web version of this article.)

The measured population-level distributions and the corresponding lognormal fits are shown in Fig. 2 for the control wells (not containing ChrABC) and for the biosensor wells containing 10^−4^ U/ml and 1 U/ml concentrations of ChrABC during the cell adhesion process. The 1 U/ml ChrABC concentration is especially interesting since Moyano et al. [47] and Lee et al. [48] also studied cancer cells line (TE-1 cell line) at this value. Moyano et al. reported an adhesion decreasing effect of the treatment, claiming that integrins could not effectively bind to fibronectin. In contrast, Lee et al. reported an increase in cell adhesion. In our previous work, we found a decrease in adhesion for 1 U/ml ChrABC treatment when examining the averaged behavior of large cell populations (8000 cells). In contrast, 10^−4^ U/ml ChrABC slightly increased the adhesion strength and speed 1. Note, this concentration range has been previously studied by WGA lectin staining to visualize the various components of glycocalyx and fluorescence intensity was measured (see SI Fig. S3-4 in ref. 1).

All together 100–120 individual cells were measured and analyzed for every concentration. The plotted distributions are corresponding to the 90 min signals after cell addition to the wells, basically at the saturation level of the adhesion process at these conditions [1], [28]. It is clearly seen in Fig. 2 that the adhesivity distributions are well fitted by the lognormal distribution in all cases. The digestion has an interesting effect on the adhesivity distributions, yet hidden when investigating population averages. At 10^−4^ U/ml concentration a small population appears with large adhesivity. In contrast, at the 1 U/ml ChrABC concentration a distinct subpopulation with a very low adhesivity signal is clearly observable (see black arrows in Fig. 2).

**Fig. 3:**
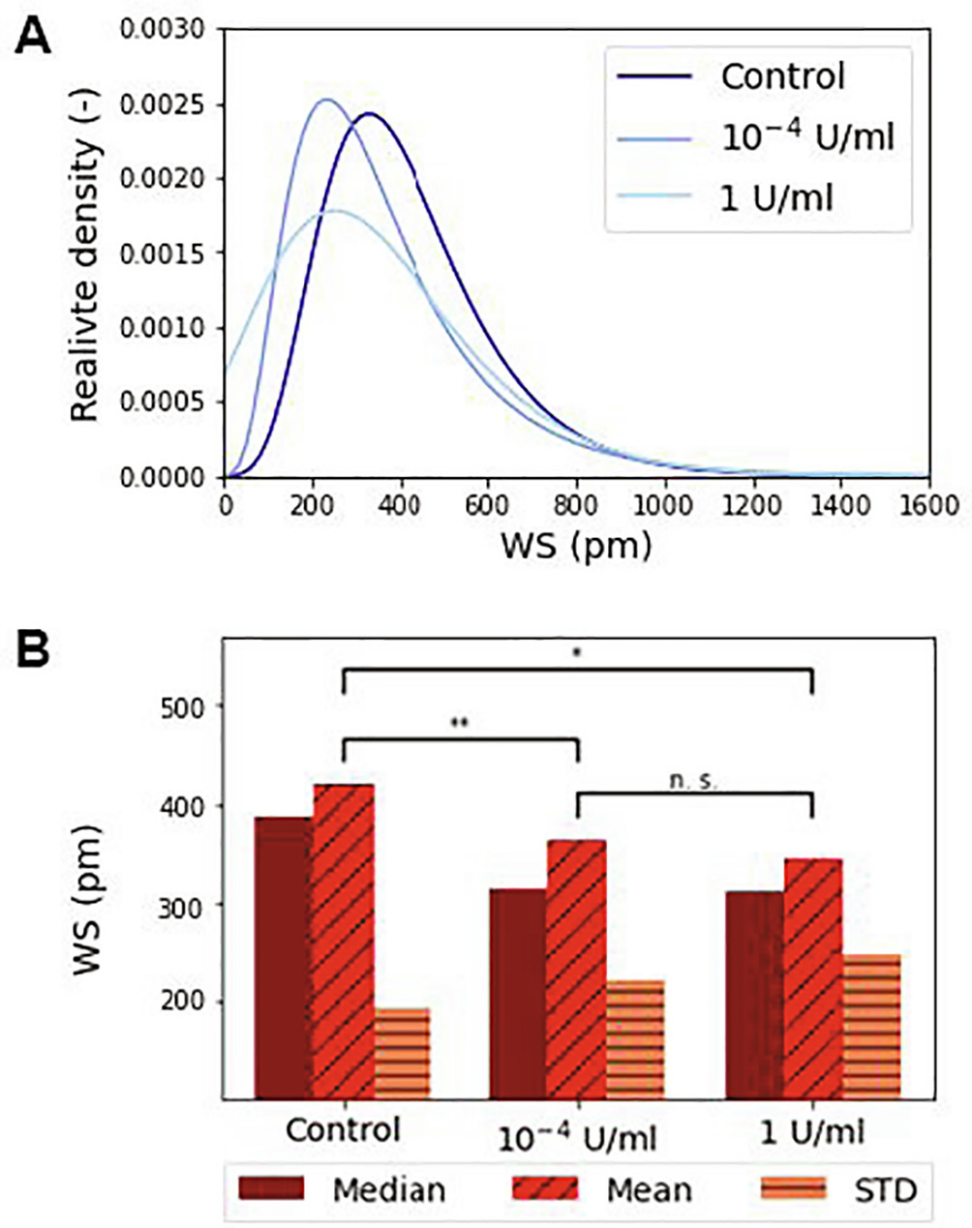
A The lognormal fits of population distributions of single-cell adhesivity at various ChrABC concentrations. B The mean, median and standard deviations (STD) of the fitted distributions are also shown. A changing trend due to enzyme treatment is clearly visible with significance levels indicated. *p < 0.05, **p < 0.01. The Kruskal-Wallis H-test test with Wilcoxon signed-rank post-test was employed.

The changes in the fitted distributions are further emphasized in Fig. 3. Due to the enzyme treatment the mean and median values of the distributions are decreased, but the standard deviation follows an opposite trend. The digestion of the glycocalyx widens the adhesivity distribution, cell heterogeneity clearly increases with enzyme treatment.

Knutson et al. reported that chondroitin sulfate can improve integrin function during their binding to ligands [49]. Paszek et al. reported that glycocalyx mediates the integrin-ligand interaction to a large extent and may therefore be a functional cytostatic regulator of integrins. The thickness and stiffness of the glycocalyx may regulate this relationship. A thick and rigid glycocalyx layer can promote integrin clustering. Note, this finding is consistent with our experimental observations [22].

Many adhesion molecules are known, including integrins, cadherins, and selectins [38], [50]. Of these, for example, cadherin-based cell adhesion can produce a fairly strong adhesive contact [51], but the effect of glycocalyx components on these contacts was not investigated in earlier works.

The enzyme ChrABC was used to digest specific glycocalyx components during adhesion by other authors, too. Some found that enzymatic digestion of cells decreased their adhesivity already at 10^−2^ U/ml ChrABC concentration [15], [47], [49], [52], [53], but the effect was not investigated at the single-cell level and no population distributions of cell adhesion are presented in any of these prior works.

Denholm et al. studied the proliferation and invasion of melanoma cells. Cancer cells were treated with ChrABC and more than 50% inhibition was reported with 10 U/ml ChrABC treatment. It has been found that the chondroitin sulfate and dermatan sulfate molecules digested by ChrABC can bind to the CD44 membrane protein, which is an important adhesion molecule, influencing cell proliferation [15], [54]. Henke and Faasen also reported the effect of ChrABC on tumor proliferation and adhesion. An adhesion reduction were observed due to the digestion of glycocalyx [55], [56].

In contrast, interestingly, Lee et al. found that the 1 U/ml ChrABC enzymatic digested TE-1 cells adhered more strongly [48]. During their measurements, the adhesion force was measured with a hydraulic micromanipulator. This observation is not supported by our findings, at this concentration we measured adhesion weakening.

**Fig. 4:**
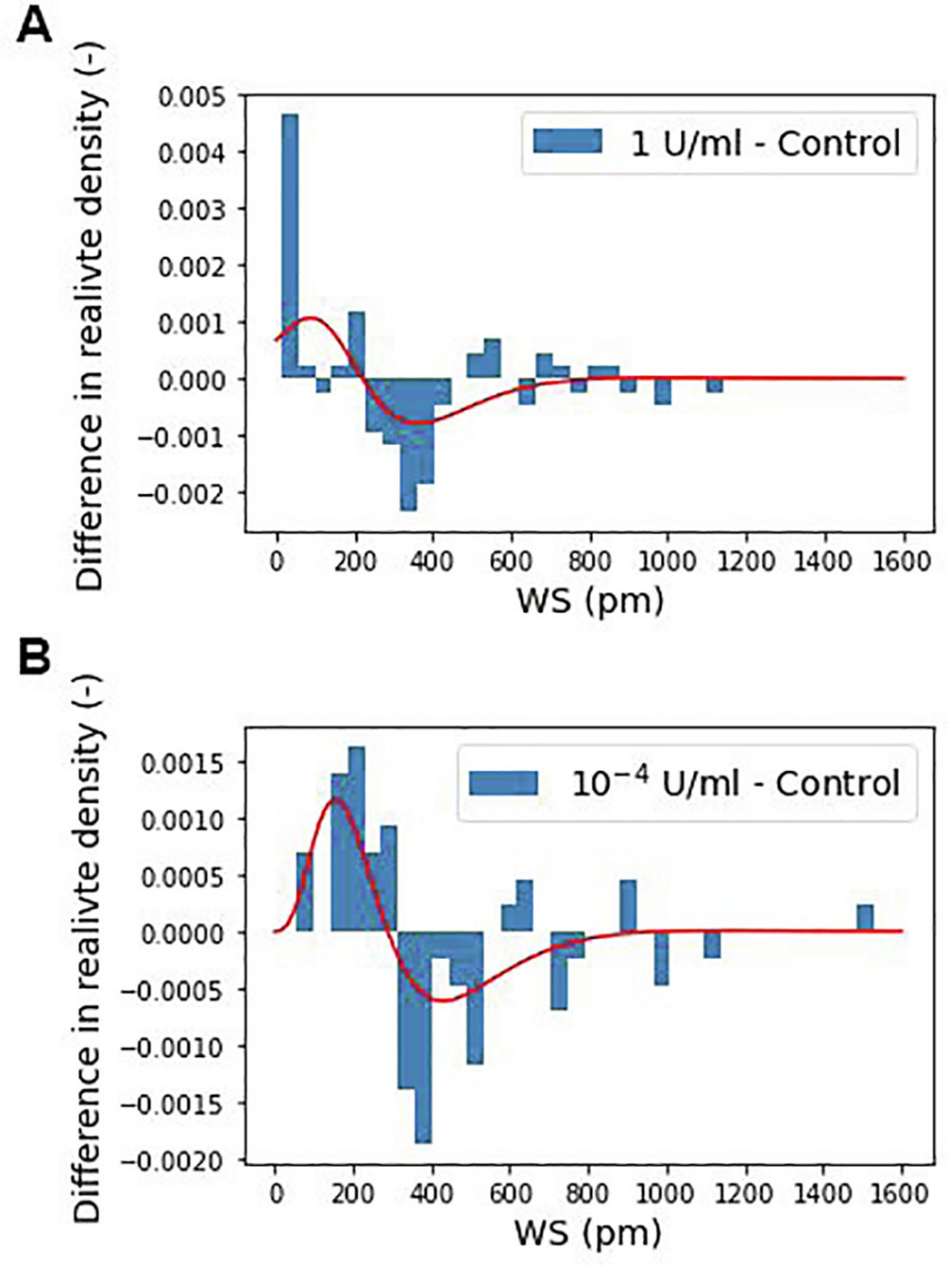
The difference in cell population behavior for A 1 U/ml and B 10^−4^ U/ml enzyme concentrations. The figures show the subtracted control population relative density bin values from the enzyme treated bin values in the function of the recorded single-cell WS values. The red line represents the difference in the fitted lognormal distribution curves to better indicate the changes due to the treatment. (Enzyme treated minus control.) (For interpretation of the references to colour in this figure legend, the reader is referred to the web version of this article.)

The subpopulation behavior after the enzyme treatment is highlighted in Fig. 4, where the relative density value of every bin of the control population was subtracted from the enzyme treated values. In this figure the positive value means an emerging, while the negative value represents a disappearing subpopulation behavior due to the ChrABC treatment. It is seen that the enzyme treatment mainly effects cells with WS value between 200 and 500 pm and here a strong decrease is seen. However, a concentration dependent behavior is observed for cells adhering with 0–200 pm values. For the larger concentration a significant number of cells with basically zero adherence appear (see Fig. 4A). In contrast, at 10^−4^ U/ml concentration here a relatively broad subpopulation behavior is emerging and no cells with practically zero adherence are seen after the treatment (Fig. 4B).

## Conclusions and outlook

Cancer cells have a thickened glycocalyx layer compared to healthy cells, but still, there is no consensus in the literature on its exact role in cellular adhesion and signaling. In our previous work, we showed that different degrees of digestion of the glycocalyx of cancer cells influence the kinetics and strength of adhesion on coatings with RGD peptide motifs. Depending on the employed ChrABC concentration, adhesion strengthening and weakening could be observed considering the averaged behavior of 8000 cells. The effect of the enzyme was also examined with MCF-7 cancer cells and a preosteoblast healthy cell line, but it did not show a strong adhesion-reducing effect [1]. In the present study, a high spatial resolution resonant waveguide grating biosensor was employed to monitor the adhesivity of HeLa cancer cells at the single-cell level for hundreds of cells. Note, this technique could be also employed to measure the adhesivity of confluent cell layers, for example the adhesivity of endothelial layers, to investigate lateral inhomogeneity inside the layer with excellent sensitivity. But due to the limited lateral resolution, the identification of individual cells would be more challenging by using the biosensor images only. Our aim was to reveal the population-level distributions of cell adhesivity when ChrABC was added to the adhering cells. We found that the population distributions of adhesivity can be well fitted by lognormal distributions. Enzymatic digestion of glycocalyx resulted in a broader population distribution with significantly lower mean and median values. Moreover, at relatively low and high enzyme concentrations, subpopulations with remarkably large and weak adhesivity were also identified. Digestion of the cell with ChrABC yielded wider population distributions than in the undigested case and a significant difference was obtained. To our knowledge, this work is the first where enzymatic digestion of glycocalyx components of cancer cells was studied in population distribution relation.

By 2020, nearly 10 million people worldwide will have lost the fight against cancer and about 19.3 million new diseases have been diagnosed, with projections predicting 28.4 million new diseases by 2040. Based on these, it is no exaggeration to say that one of the most important health tasks today is to find a way to overcome as many cancers as possible [57]. Previous works mainly investigated cell population averages [58], [59], [60]. This work highlights the importance of single-cell adhesion assays in cancer research. The results can have far-reaching implications in drug development processes, as drugs can be developed for specific subpopulations of cancer cells. We showed that cells originally adhering in the 200–500 pm WS range were most influenced by the ChrABC treatment. In future works these cells could be selected by single cell manipulation techniques [36], [37] and investigated further in order to potentially develop more reliable and meaningful cancer drugs affecting adhesion.

### CRediT authorship contribution statement

**N. Kanyo:** Data curation, Writing – original draft. **K.D. Kovács:** Software, Writing – review & editing. **S.V. Kovács:** Data curation. **B. Béres:** Software. **B. Peter:** Visualization, Writing – review & editing. **I. Székács:** Conceptualization, Methodology, Writing – review & editing. **R. Horvath:** Conceptualization, Methodology, Supervision, Writing – review & editing.

## Declaration of Competing Interest

The authors declare that they have no known competing financial interests or personal relationships that could have appeared to influence the work reported in this paper.
